# Inhibitors targeting the autophosphorylation of serine/threonine kinase of *Streptococcus suis* show potent antimicrobial activity

**DOI:** 10.3389/fmicb.2022.990091

**Published:** 2022-09-02

**Authors:** Haotian Li, Tingting Li, Qiao Hu, Zhiming Yao, Lu Li, Qi Huang, Rui Zhou

**Affiliations:** ^1^State Key Laboratory of Agricultural Microbiology, College of Veterinary Medicine, Huazhong Agricultural University, Wuhan, China; ^2^Cooperative Innovation Center of Sustainable Pig Production, Wuhan, China; ^3^International Research Center for Animal Disease (Ministry of Science & Technology of China), Wuhan, China; ^4^The HZAU-HVSEN Institute, Wuhan, China

**Keywords:** serine/threonine kinase, antimicrobial, drug target, autophosphorylation, inhibitor, *Streptococcus suis*

## Abstract

Antimicrobial resistance (AMR) is a global concern threatening public health. Developing novel antibiotics is one of the effective strategies to tackle AMR. Serine/threonine kinases (STKs) have been recently shown to play critical roles in the physiology and pathogenesis of several important bacterial pathogens which are regarded as a promising antimicrobial drug target. We previously reported the roles of STK in the regulation of bacterial cell division, metabolism, and pathogenesis in *Streptococcus suis*, an important zoonotic bacterial pathogen. In this study, we firstly identified the Thr167 and Ser175 residues in the activation loop of *S. suis* STK (ssSTK) as the kinase autophosphorylation sites. Phenotyping results demonstrated that the autophosphorylation deficient strain resembled the *stk* deletion strain showing essentiality for bacterial growth in minimal medium, abnormal morphology, and decreased virulence when compared with the wild-type *S. suis* SC19 strain. Based on these findings, we established an ssSTK inhibitor screening approach by measuring the growth of *S. suis* in a minimal medium and testing the autophosphorylation inhibition by measuring the consumption of ATP in an enzymatic reaction by ssSTK. A series of inhibitors against ssSTK are identified from a commercial kinase inhibitors library, including Staurosporine, K252a, AT9283, and APY29. These inhibitors showed antimicrobial activity *in vitro*. Moreover, by using *Galleria mellonella* larvae infection assay, compound APY29 displayed *in vivo* efficacy against *S. suis* infection. Additionally, it was predicted by molecular docking that these inhibitors could interact with ssSTK. Collectively, our data illustrated the essential roles of ssSTK autophosphorylation in the physiology and pathogenicity of *S. suis* and consider these inhibitors as promising antimicrobial lead compounds.

## Introduction

Antimicrobial resistance (AMR) has become a serious problem that poses a huge threat to public health and animal husbandry worldwide (Samreen et al., 2021). It is listed as one of the top 10 threats to global health by the World Health Organization (WHO) in 2019, which causes at least 700,000 deaths every year (Mancuso et al., 2021). Great efforts have been made to tackle AMR. These include the implementation of administrative policies to promote the rational use of antibiotics (Gyssens, 2011; Xiao et al., 2013), development of antibiotics alternatives such as vaccines, probiotics, antimicrobial peptides, and phage therapy to reduce the use of antibiotics (Allen et al., 2014; Hoelzer et al., 2018; Browne et al., 2020). On the other hand, in the past 30 years, few novel classes of antibiotics have been developed, which is also one of the reasons leading to the accumulation of AMR (Lewis, 2020). Therefore, another important aspect to combat AMR is to accelerate the development of novel antibiotics.

Protein phosphorylation is an important posttranslational modification in prokaryotic and eukaryotic cells that participates in regulating a wide variety of cellular processes (Sun et al., 2015; Mijakovic et al., 2016; Lavoie et al., 2020). The critical role of protein phosphorylation makes protein kinases attractive drug targets (Chen et al., 2015). Until 2021, the United States Food and Drug Administration (FDA) has approved 71 small-molecule kinase inhibitors for treating a variety of diseases including tumors and non-malignancies (Roskoski, 2021). However, so far, there have been no antimicrobials developed that target bacterial kinases.

Important roles of reversible protein phosphorylation mediated by bacterial kinases and phosphatases have been noted in the past decades and several bacterial protein kinases have been proposed as potential targets for novel antibiotics (King and Blackledge, 2021). The two-component systems (TCSs) are the well-characterized protein phosphorylation systems that play important roles in sensing and responding to environmental signals (Alvarez et al., 2016). They are composed of histidine kinases (HKs) and response regulators (RRs) that are involved in the regulation of bacterial virulence, biofilm formation, and antimicrobial resistance (Zschiedrich et al., 2016; Tierney and Rather, 2019; Isaka et al., 2021). Inhibitors against the TCSs have been identified to have anti-virulence (Rasko et al., 2008) and even antimicrobial activity (Velikova et al., 2016a).

Recently, the serine/threonine kinases (STKs) have been shown as important regulators in several important bacterial pathogens, including *Mycobacterium tuberculosis*, *Staphylococcus aureus*, *Listeria monocytogenes*, *Streptococcus pneumoniae*, and *Streptococcus suis* (Débarbouillé et al., 2009; Osaki et al., 2009; Chawla et al., 2014; Pensinger et al., 2014; Zhu et al., 2014; Manuse et al., 2016; Nagarajan et al., 2021). STKs usually harbor an extracellular penicillin-binding protein and serine/threonine kinase associated (PASTA) domain and an intracellular catalytic kinase domain (Manuse et al., 2016). In response to stimuli, STK autophosphorylates on its activation loop of the kinase domain by hydrolyzing adenosine triphosphate (ATP) and transfers the phosphate to the serine or threonine residues of its substrate proteins (Ogawara, 2016; Labbe and Kristich, 2017; Nagarajan et al., 2021). STKs are identified as global regulators controlling a wide range of important bacterial physiological processes, including cell wall hemostasis, virulence, metabolism, and cell division in several important bacterial pathogens (Cheung and Duclos, 2012; Chawla et al., 2014; Manuse et al., 2016; Wamp et al., 2020; Djorić et al., 2021). In some bacteria, STKs are even essential or conditional essential for bacterial viability (Fernandez et al., 2006; Hu et al., 2021). Based on their critical physiological roles, several STK inhibitors have been screened to have antimicrobial activity (Fernandez et al., 2006; Wehenkel et al., 2006; Maslov et al., 2019) or as antibiotic adjuvants to potentiate *β*-lactam activity (Schaenzer et al., 2017, 2018).

*Streptococcus suis* is a zoonotic bacterial pathogen that causes lethal infections in pigs and humans (Lun et al., 2007; Tan et al., 2019). Currently, antibiotics are still the major approach to treat the disease caused by *S. suis*. However, it is more difficult to control *S. suis* infections due to the rapid emergence of AMR (Devi et al., 2017; Oh et al., 2017; Tan et al., 2021). Thus, developing novel antibiotics is of great importance in controlling *S. suis* infection. *Streptococcus suis* only harbors one copy of the gene encoding STK. Several studies have revealed that ssSTK plays an important role in the growth, metabolism, and pathogenesis of *S. suis* (Zhu et al., 2014; Zhang et al., 2017; Hu et al., 2021; Liu et al., 2021). More importantly, our previous study has demonstrated that disruption of *stk* almost completely abolished the growth of *S. suis* in the minimal medium (Hu et al., 2021).

In this study, we identified the autophosphorylation sites of ssSTK in its activation loop and characterized the key role of ssSTK autophosphorylation in the growth, morphology, and virulence of *S. suis*. Based on the essentiality of ssSTK autophosphorylation for cell growth in the minimal medium as well as by establishing an enzymatic assay to test the autophosphorylation inhibition by measuring the consumption of ATP, four compounds are identified as potent inhibitors against ssSTK by high throughput screening from a drug library with compound analogous characterization. These inhibitors showed strong inhibition against *S. suis* growth in the minimal medium and one of them showed *in vivo* antimicrobial activity in the *Galleria mellonella* larvae infection assay. Our study identifies effective lead compounds by screening STK inhibitors which provide good candidates for novel antimicrobials development.

## Materials and methods

### Bacterial strains and growth conditions

All bacterial strains used in this study are listed in Supplementary Table S1. *Streptococcus suis* SC19 is a virulent serotype 2 strain isolated in a diseased pig during an outbreak in Sichuan Province, China, in 2005 (Lun et al., 2007). *Streptococcus suis* SC19 and its derived strains were grown at 37°C in tryptic soy agar (TSA) or tryptic soy broth (TSB) medium supplemented with 10% fetal bovine serum (FBS, Cat#23022-8615, Every green, Hangzhou, China) or the chemically defined medium (CDM) prepared as previously described (van de Rijn and Kessler, 1980) of which the composition is listed in Supplementary Table S2. The *stk*-deleted strain of *S. suis* SC19 (Δ*stk*) and its complementary strain (CΔ*stk*) were constructed in our previous study (Hu et al., 2021). The *S. suis* Δ*stk::stk*^T167A-S175A^ strain was constructed by introducing a pSET2-based plasmid into the Δ*stk* strain to express the variant STK (T167A-S175A point mutations) *in trans*. *Escherichia coli* DH5α strain was used as the host strain for regular cloning. *E. coli* MC1061 strain was used as the host strain for cloning of pSET2-derived plasmids. *E. coli* BL21(DE3) strain was used as the host strain for pET28a-derived plasmids for protein expression and purification.

### Plasmids construction

The plasmids used in this study are listed in Supplementary Table S1. Plasmid pSET2-*pro-stk*^T167A-S175A^ encodes the variant ssSTK (T167A-S175A point mutations) in *S. suis* in which the native promoter of *stk* was used to drive the transcription of the variant *stk* genes in *S. suis*. The promoter region and the coding sequence of the *stk* containing the autophosphorylation site mutations were amplified and subsequently cloned into pSET2 vector, resulting in plasmid pSET2-*pro-stk*^T167A-S175^. The coding sequences of the kinase domain of ssSTK and its variants ssSTK^KD-T167A^, ssSTK^KD-T169A^, and ssSTK^KD-S175A^ were amplified by overlap extension PCR. The PCR products were then cloned into the pET28a vector to generate the recombinant expression plasmids pET28a-*stk^KD^*, pET28a-*stk^KD-T167A^*, pET28a-*stk^KD-T169A^*, and pET28a-*stk^KD-S175A^*. All the primers used in this study are listed in Supplementary Table S3.

### Protein expression and purification

The 6 × his-tagged recombinant proteins were expressed and purified as previously described (Zhang et al., 2017). Briefly, *E. coli* BL21 (DE3) containing the pET28a-*stk^KD^*, pET28a-*stk^KD-T167A^*, pET28a-*stk^KD-T169A^*, or pET28a-*stk^KD-S175A^* plasmid was grown to the mid-log phase and protein expression was induced by the addition of 1 mM isopropyl-β-D-thiogalactopyranoside (IPTG) followed by incubation at 18°C for 16 h. The cells were harvested and lysed by sonication. The cell lysate was subjected to centrifugation at 12,000 rpm for 10 min at 4°C to remove the unbroken cells and cell debris. The supernatant was then subjected to affinity chromatography with the Ni-NTA column (Cat# 10271899, GE Healthcare, Uppsala, Sweden). The purified protein was concentrated by ultrafiltration and stored at −80°C until use.

### Virulence assay using a *Galleria mellonella* larvae model

*Galleria mellonella* larvae model was used to evaluate the virulence of *S. suis* as previously described (Velikova et al., 2016b). A total of 50 *G. mellonella* larvae were randomly divided into five groups (10 per group), which were injected with 1 × 10^6^ CFU of *S. suis* SC19, Δ*stk*, CΔ*stk*, Δ*stk::stk^T167A-S175A^*, or the same volume of saline, respectively, *via* the left posterior proleg of *G. mellonalla* larvae. The survival of *G. mellonella* larvae was recorded at 6 h intervals for 78 h. *In vivo* assessment of antimicrobial activity of APY29 using *G. mellonalla* larvae infection model was performed as follows. 1 × 10^6^ CFU of *S. suis* SC19 were injected into the left posterior proleg of *G. mellonalla* larvae (10 per group), followed by injection of different doses of APY29, respectively. A control group was set, in which saline was injected and without *S. suis* SC19 infection (Mock). The survival of *G. mellonella* larvae was recorded at 6 h intervals for 48 h.

### High-throughput screening for ssSTK inhibitors

A commercial kinase inhibitors library (HY-LD-000001801), containing 1,133 compounds was bought from MedChemExpress (MCE), and the detailed information is listed in Supplementary Table S5. To screen for inhibitors against ssSTK, the mid-log phase cells of *S. suis* SC19 and Δ*stk* were diluted in CDM in 96-well plates containing either the library compounds or the same volume of dimethyl sulfoxide (DMSO). After 8 h of static culture at 37°C, the optical density at 600 nm (OD_600_) was measured using a microplate spectrophotometer (FLUOSTAR OMEGA, BMG LABTECH). Percent inhibition was calculated as (OD_X_ – OD_N_)/(OD_P_ – OD_N_) × 100%, where OD_X_ is the OD_600_ value for a test treated with compound X, and OD_P_ and OD_N_ are the OD_600_ values for SC19 and Δ*stk* treated with DMSO, respectively.

### *In vitro* ssSTK autophosphorylation assay

ssSTK phosphorylates itself by hydrolyzing ATP. Therefore, the autophosphorylation activity can be determined by measuring the consumption of ATP. The autophosphorylation assays were carried out in 50 μl of kinase reaction buffer (HEPES 50 mM, DTT 1 mM, Brij35 0.01%, pH 7) containing 10 mM MgCl_2_, 100 μM ATP, 10 μM ssSTK, and 0.5 μl of compound or DMSO. The mixture was incubated at 37°C for 30 min. Then, 50 μl of Kinase Glo^®^ reagent (Cat# V3771, Promega, Madison, United States) was added to each well. After 10 min, the relative light unit (RLU) values were measured by using a microplate spectrophotometer. Percent inhibition was calculated as ((RLU_X_ − RLU_P_)/(RLU_N_ − RLU_P_)) × 100%, where RLU_X_ is the RLU value for a test treated with compound X, and RLU_P_ and RLU_N_ are the RLU values for the reaction mixture without the treatment of compound and the reaction mixture lacking ssSTK, respectively. Additionally, the autophosphorylation activity is also determined by measuring its ssSTK autophosphorylation levels after the enzymatic reaction. The mixture (kinase buffer, 10 mM MgCl_2_, 50 μM ATP, and 2 μM ssSTK) was supplemented with Staurosporine, K252a, AT9283, and APY29 at 10, 50, 100 μM or DMSO, respectively. Then, these samples were incubated at 37°C for 30 min and terminated by the addition of 6 × SDS loading buffer. Subsequently, the sample was boiled for 10 min and analyzed by SDS-PAGE. The protein was transferred to the polyvinylidene difluoride (PVDF) membrane and the phosphorylated protein was probed by an anti-P-Threonine mouse monoclonal antibody (Cat# 9386S, Cell Signaling, Boston, United States) diluted at 1:2,000 followed by incubation with the HPR-conjugated anti-mouse IgG antibody (Cat# SA00001-1, Proteintech, Wuhan, China) diluted at 1:4,000 in PBS with 5% BSA as the secondary antibody. Then, the phosphorylated protein was visualized by autoradiography.

### Determination of half-maximal inhibitory concentration of inhibitors against ssSTK

The reaction was conducted in a 50 μl mixture containing 10 μM ssSTK in the kinase reaction buffer supplemented with 0.5 μl of DMSO or Staurosporine, APY29, AT9283, and K252a with final concentrations ranging from 0 μM to 100 μM in a 96-well black plate, respectively. The enzymatic inhibition percentage was calculated as described above. The data were transformed to log scale and non-linear regression was performed with GraphPad Prism software (version 7) using the variable slope normalized model for enzyme inhibition to determine IC_50_.

### Bacterial growth assay

Cells of *S. suis* SC19, Δ*stk*, CΔ*stk*, and Δ*stk::stk*^T167A-S175A^ were grown to the mid-log phase in TSB or CDM, respectively. The cells were then subcultured 1:100 into the corresponding medium with or without the ssSTK inhibitors in a 100-well plate. The plate was incubated at 37°C with shaking and the growth was monitored using an automatic growth curve analyzer (Oy Growth Curves Ab Ltd., Helsingfors, Finland).

### Gram staining

Cells of *S. suis* were grown to the mid-log phase in the TSB medium. Subsequently, the cells were washed twice and resuspended in saline. A loop of bacterial cells was fixed on glass slides (Shitai, China) through flaming. A Gram staining kit (Cat#D008, Jiancheng, China) was utilized according to the manufacturer’s instructions. The stained samples were observed using an optical microscope (OLYMPUS).

### *In silico* docking

The 3D structure of the kinase domain of ssSTK was predicted using the I-TASSER server^1^ (Roy et al., 2010; Yang et al., 2015; Yang and Zhang, 2015). The complex models of the kinase domain of ssSTK with inhibitors were generated using autodock4 software. The interacting models were displayed by using PyMOL (version 2.0.6.0) or Ligplus software (version 2.2; Laskowski and Swindells, 2011).

### Statistical analysis

The data were commonly analyzed by a two-tailed Student’s *t*-test in GraphPad Prism 7 software, with a value of *p* < 0.05 considered to be statistically significant. The survival data were analyzed by log−rank test in GraphPad Prism 7 software, with a value of *p* < 0.05 considered to be statistically significant.

## Results

### Identification of STK autophosphorylation sites

**Figure 1:**
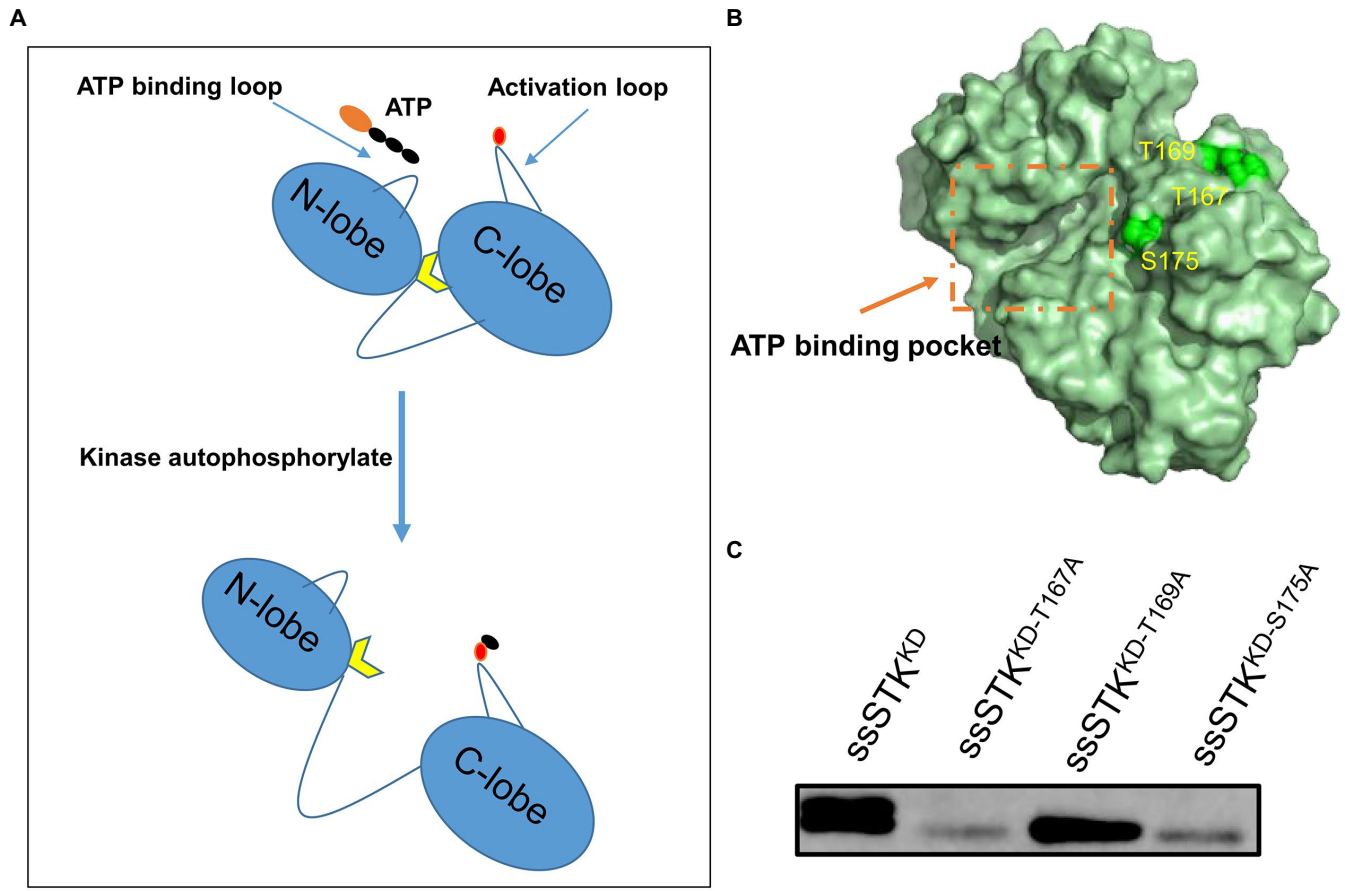
Identification of autophosphorylation sites of ssSTK. **(A)** Schematic diagram of the process of autophosphorylation of STK. The autophosphorylation sites of the kinase activation loop are phosphorylated, which exposes the substrate binding sites. Black ellipsoid denotes phosphate group; Red ellipsoid denotes the autophosphorylation sites of kinase activation loop; Yellow arrow denotes the substrate binding sites. **(B)** The simulated 3D structure of ssSTK. The amino acid sequence of ssSTK was analyzed using the I-TASSER server. The image was generated by PyMOL software. Green spheres represent the predicted autophosphorylation sites of ssSTK. **(C)** The autophosphorylation assay. Each purified protein (ssSTK^KD^, ssSTK^KD-T167A^, ssSTK^KD-T169A^, and ssSTK^KD-S175A^) was added to kinase buffer including 50 μM ATP, respectively. Then, the mixtures were incubated at 37°C for 30 min and terminated by the addition of SDS loading buffer. The autophosphorylation levels of these samples were measured by western blotting using an anti-P-Threonine mouse monoclonal antibody; The experiment was carried out two times independently under the same conditions.

STK contains an activation loop where autophosphorylation occurs which promotes the exposure of the substrate binding sites (Figure 1A). Previous studies have identified the autophosphorylation sites of other bacterial STK (Durán et al., 2005; Tomono et al., 2006; Zheng et al., 2018). By homologous comparison, it was predicted that Thr167, Thr169, and Ser175 of ssSTK are the autophosphorylation sites of ssSTK (Supplementary Figure S1A), which were shown in the simulated ssSTK 3D structure (Figure 1B). To verify these sites, the kinase domain of ssSTK (ssSTK^KD^) and the domain containing each point mutation (ssSTK^KD-T167A^, ssSTK^KD-T169A^, and ssSTK^KD-S175A^) were purified, with which autophosphorylation assays were conducted (Supplementary Figure S1B). It was shown in Figure 1C and Supplementary Figure S2A that ssSTK^KD-T167A^ and ssSTK^KD-S175A^ displayed significantly decreased autophosphorylation, while the STK^KD-T169A^ showed a similar level of autophosphorylation compared with ssSTK^KD^. The results indicate that Thr167 and Ser175 are the autophosphorylation sites of ssSTK.

### Autophosphorylation of ssSTK is critical for the growth, morphology, and pathogenicity of *Streptococcus suis*

**Figure 2:**
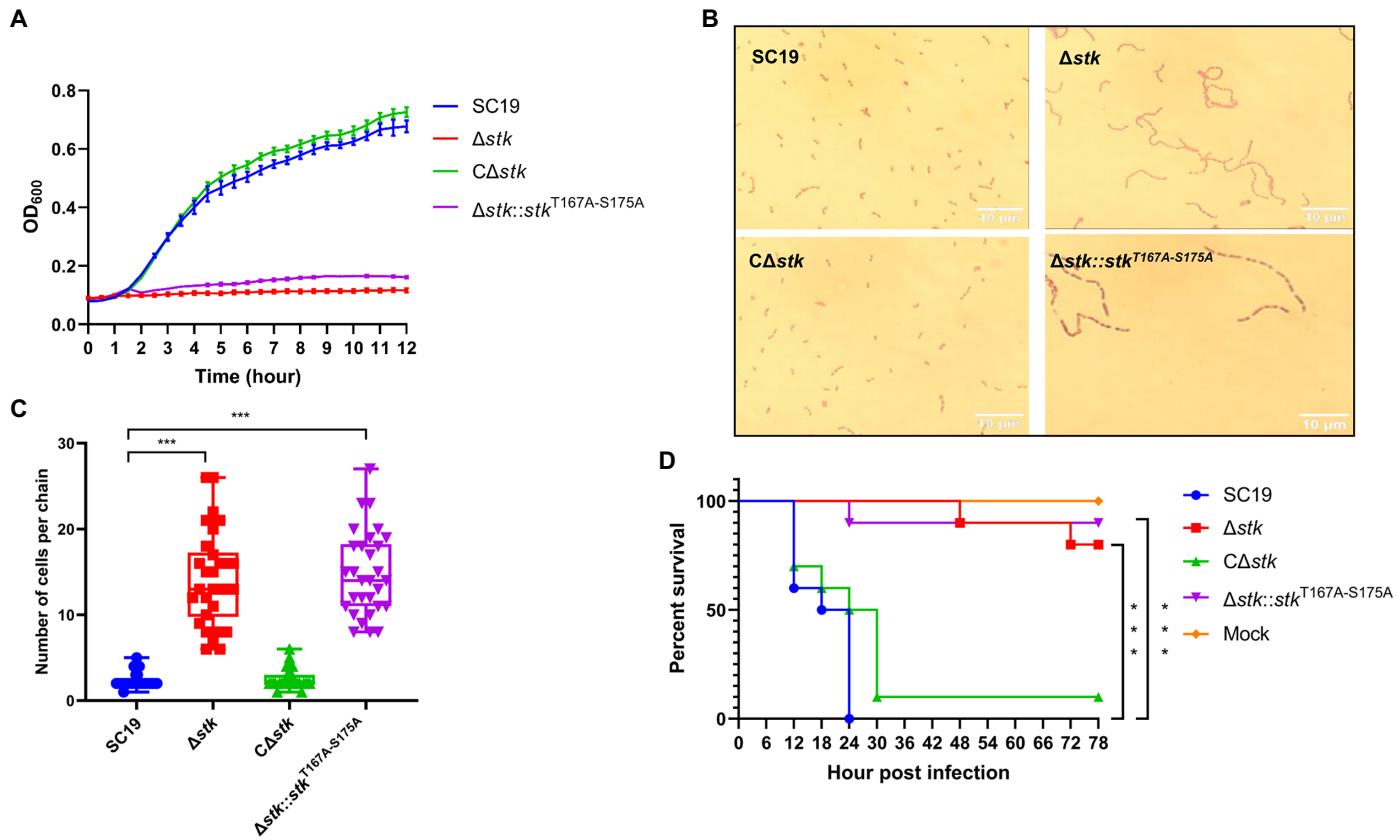
The role of autophosphorylation of ssSTK in the morphology, growth, and pathogenicity of *Streptococcus suis*. **(A)** Growth assay. Cells of *S. suis* SC19, Δ*stk*, CΔ*stk*, and Δ*stk::stk*^T167A-S175A^ were grown in the chemically defined medium (CDM) and OD_600_ monitored; **(B)** Gram staining assay. Cells of *S. suis* SC19, Δ*stk*, CΔ*stk*, and Δ*stk::stk*^T167A-S175A^ were grown to the mid-log phase. Then, the cells were washed twice and resuspended in normal saline. Each sample (20 μl) was fixed on glass slides and stained. The samples were then observed using an optical microscope. **(C)** 30 chains were randomly chosen from each Gram staining images of SC19, Δ*stk*, CΔ*stk,* and Δ*stk*::*stk*^T167A-S175A^, respectively. Next, numbers of cells per chain were counted, respectively, and the data were presented as the means ± standard errors (*n* = 30). Student’s *t*-test was used to analyze their chain length difference in GraphPad Prism 7 software, with the value of *p* < 0.001, ***. **(D)** *G. mellonella* larvae infection assay. The *G. mellonella* larvae were inoculated with 1 × 10^6^ CFU of *S. suis* SC19, Δ*stk*, CΔ*stk*, Δ*stk::stk*^T167A-S175A^ and the same volume saline (Mock). The survival for each group was recorded every 6 h for 78 h. The survival data were analyzed by log−rank test in GraphPad Prism 7 software, with the value of *p* < 0.001, ***. The experiments were carried out two times independently under the same conditions.

Our previous studies revealed that deletion of *stk* in *S. suis* resulted in growth defects, abnormal morphology, and decreased virulence (Zhang et al., 2017). To further investigate the role of autophosphorylation of ssSTK in these processes, we constructed an *S. suis* strain Δ*stk::stk*^T167A-S175A^ which expresses ssSTK with the autophosphorylation sites substituted with alanine. By performing a growth assay, it was revealed that the growth of wild-type *S. suis* SC19 strain and the CΔ*stk*, a strain expressing STK in Δ*stk*, grew faster than Δ*stk* and Δ*stk::stk*^T167A-S175A^ strains (Figure 2A). The Gram staining assay showed that SC19 and CΔ*stk* strains exhibited a normal chain length which contained 2–5 cells per chain, while Δ*stk* and Δ*stk::stk*^T167A-S175A^ cells formed significantly longer chains (Figures 2B,C). By using a *G. mellonella* larvae infection model, the pathogenicity of the *S. suis* strains was evaluated. It was shown that after infection the *G. mellonella* larvae infected with SC19 and CΔ*stk* strains died rapidly. In contrast, the percentage of survived *G. mellonella* larvae injected with Δ*stk* or Δ*stk::stk*^T167A-S175A^ was significantly higher than those injected with SC19 strain (Figure 2D). Collectively, our data suggest that ssSTK autophosphorylation plays an important role in growth, cell division, and pathogenesis of *S. suis*.

### Screening of inhibitors targeting STK of *Streptococcus suis*

**Figure 3:**
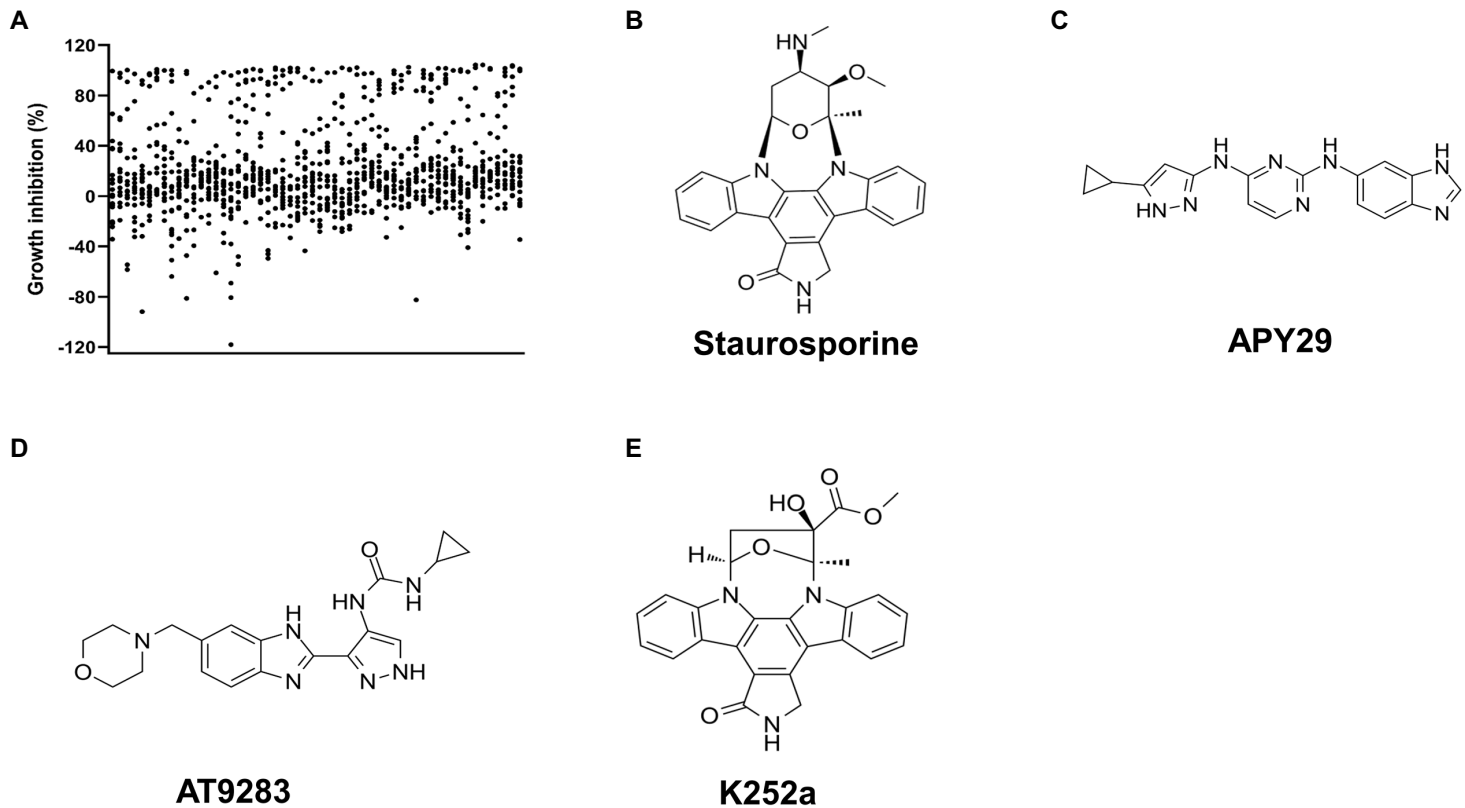
Screening of ssSTK inhibitors. **(A)** The scatter plot of the primary screening with the 1,133 compounds; **(B–E)** The structure of Staurosporine, APY29, AT9283, and K252a, respectively.

We next performed inhibitors screening against ssSTK. Since disruption of either the whole STK or its autophosphorylation sites results in abolished growth of *S. suis* in the minimal medium (Hu et al., 2021; Figure 2A). Primary screening was conducted by monitoring the growth of *S. suis* SC19 cells in the presence of each compound from the kinase inhibitors library in the CDM. A total of 1,133 compounds were tested among which 121 compounds showed a growth inhibition of over 80% (Figure 3A; Supplementary Table S4). However, these compounds are not necessarily targeting STK to impede the growth of *S. suis*. Therefore, we continued to measure the inhibitory activity of these 121 compounds against the autophosphorylation of ssSTK by quantifying ATP consumption using an *in vitro* enzymatic assay. Finally, three compounds Staurosporine, APY29, and AT9283 were identified as inhibitors against ssSTK, respectively (Figures 3B–D).

### Inhibition of autophosphorylation of ssSTK *in vitro*

**Figure 4:**
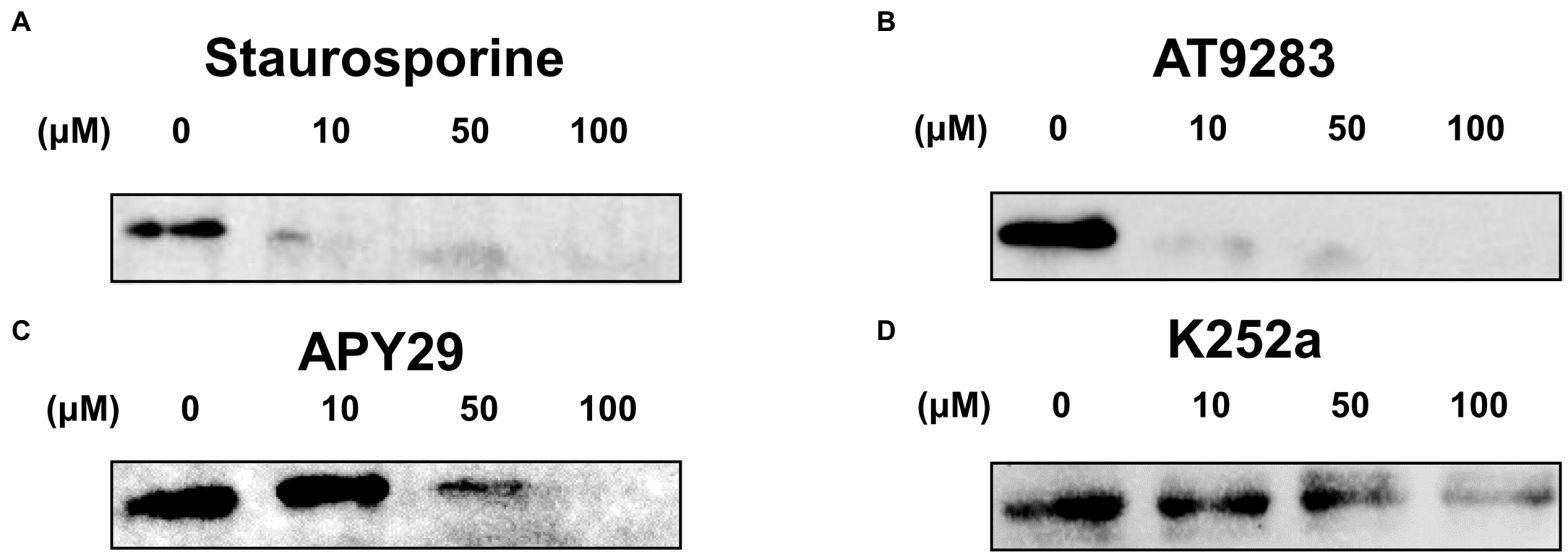
Autophosphorylation assay. Inhibitors including Staurosporine **(A)**, AT9283 **(B)**, APY29 **(C)**, and K252a **(D)** at 100, 50, 10, 0 μM, and ssSTK were co-incubated at 37°C for 30 min, respectively. Next, the samples were boiled in SDS loading buffer and were detected for the autophosphorylation of ssSTK by western blotting using anti-P-Threonine mouse monoclonal antibody. The experiments were carried out two times independently under the same conditions.

To further confirm the inhibitory activity of these inhibitors, *in vitro* autophosphorylation of ssSTK was tested in the presence of these compounds by Western blot analysis. It was shown that Staurosporine and AT9283 at 10 μM could already inhibit the autophosphorylation of ssSTK (Figures 4A,B; Supplementary Figures S2B,C). APY29 displayed a dose-dependent inhibitory effect against ssSTK (Figure 4C; Supplementary Figure S2D). These data indicate that Staurosporine, AT9283, and APY29 are potent inhibitors targeting ssSTK autophosphorylation.

### The screening for ssSTK inhibitors from the analogues of Staurosporine, APY29, and AT9283

**Figure 5:**
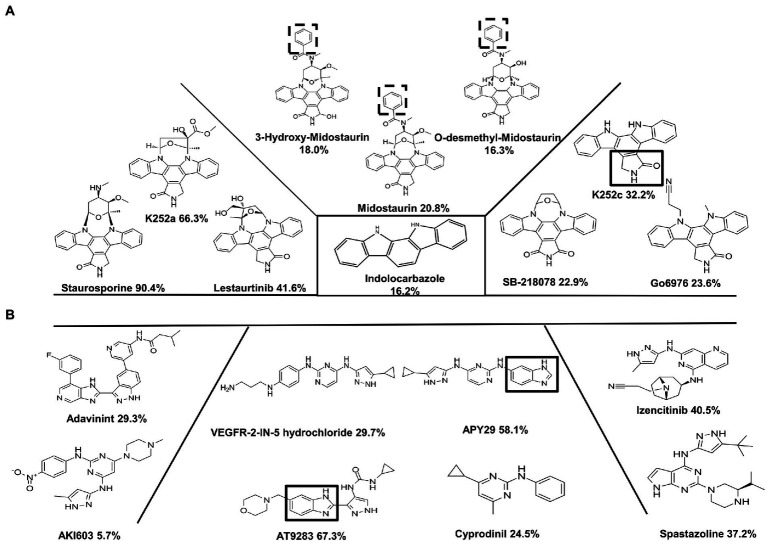
The analogs of Staurosporine, APY29, and AT9283. **(A)** The inhibition against ssSTK of Staurosporine and its analogs at 10 μM. C2-carbonyl-pyrrole ring was marked by black box. N-methybenzamide substitution at C-4′ position of pyran ring of Midostaurin, 3-Hydroxy-Midostaurin and O-desmethyl-Midostaurin were marked by black dash box. **(B)** The inhibition against ssSTK of APY29, AT9283, and its analogs at 10 μM. Benzimidazole nucleus was marked by black box. The experiments were carried out two times independently under the same conditions.

In order to discover ssSTK inhibitors with higher efficacy, analogs of these inhibitors were selected to test for their inhibitory effect against ssSTK *in vitro*. Nine Staurosporine analogs, five analogs of APY29, and one analog of AT9283 were subjected to autophosphorylation test with ssSTK (Figure 5). It was revealed that most of these analogs except K252a (Figure 3E) exhibited an inhibition lower than 50% (Figures 5A,B). Subsequently, the *in vitro* autophosphorylation assay confirmed that K252a could inhibit the autophosphorylation of ssSTK (Figure 4D; Supplementary Figure S2E). Preliminary structure–activity relationship (SAR) analysis revealed the essential role of glycosidic linkage at N-12 and N-13 position of indolocarbazole in bioactivity against ssSTK. Additionally, the structural comparison of K252c with indolocarbazole showed that the pyrrole ring with carbonyl possibly facilitates the bioactivity of indolocarbazole derivatives against ssSTK. The comparison of Staurosporine and Midostaurin or 3-Hydroxy-Midostaurin or O-desmethyl-Midostaurin suggested that N-methybenzamide substitution at C-4′ position of pyran ring possibly reduced indolocarbazole derivatives potency (Figure 5A). The structure and activity comparison of VEGF2-2-IN-5 hydrochloride with APY29 highlighted the key role of benzimidazole nucleus in APY29 (Figure 5B).

### Determination of IC_50_ of inhibitors against ssSTK

**Figure 6:**
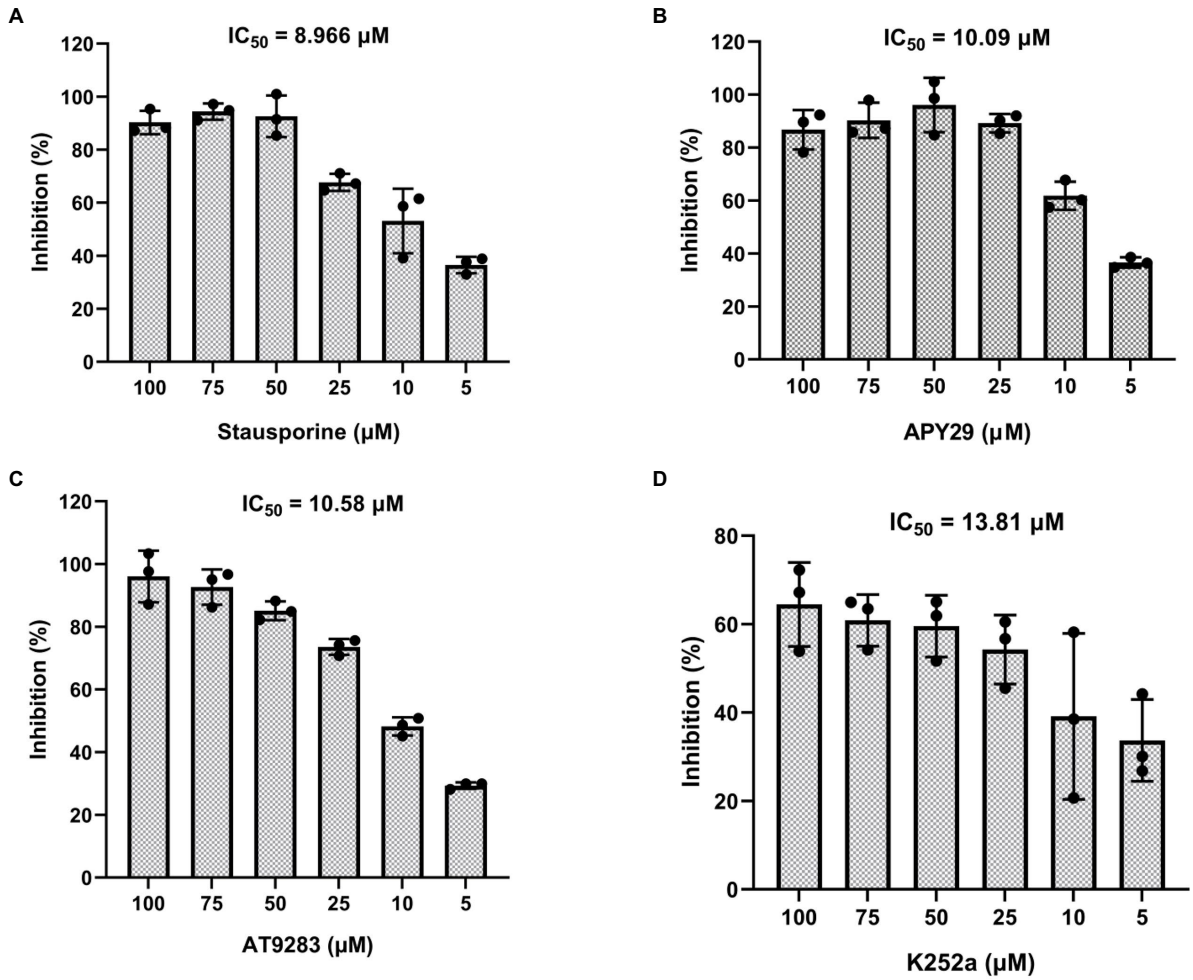
IC_50_ of inhibitors against ssSTK. Staurosporine **(A)**, APY29 **(B)**, AT9283 **(C)** and K252a **(D)** at 5, 10, 25, 50, 75, 100 μM and ssSTK were co-incubated at 37°C for 30 min, respectively. Next, the remaining ATP levels of the sample were measured by chemiluminescence technology to determine autophosphorylation levels of ssSTK. The experiments were carried out two times independently under the same conditions.

To further assess the kinetic data of Staurosporine, APY29, AT9283, and K252a, we determined the autophosphorylation activity of purified ssSTK in the presence of increasing concentrations of Staurosporine, APY29, AT9283, and K252a ranging from 0 to 100 μM. Enzymatic assays were conducted to determine the half maximal inhibitory concentration (IC_50_) of the ssSTK inhibitors. It was shown in Figure 6 that IC_50_ of Staurosporine, APY29, AT9283, and K252a against ssSTK were 8.966, 10.09, 10.58, and 13.81 μM, respectively. The data further verified the inhibitory activity of these compounds.

### *In vitro* and *in vivo* antimicrobial activity of ssSTK inhibitors

**Figure 7:**
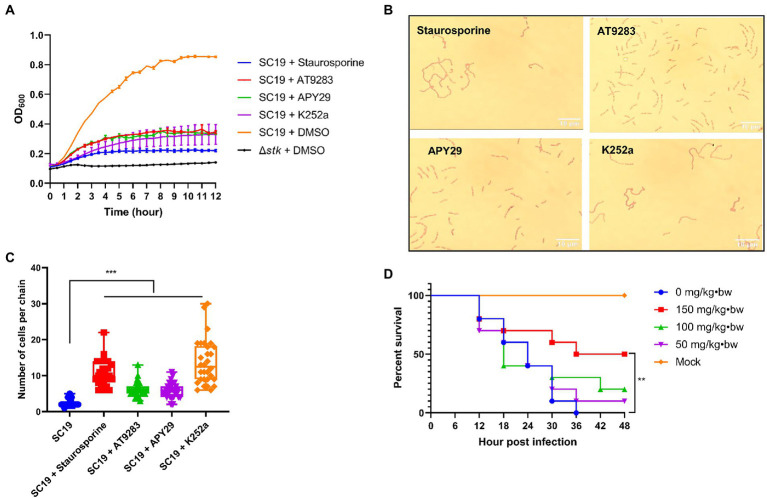
Characterizations of the STK inhibitors. **(A)** Growth assay. Cells of *S. suis* SC19 and Δ*stk* were grown in the CDM in the presence and absence of the STK inhibitors, and the OD_600_ was measured; **(B)** Influence of the STK inhibitors on the morphology of *S. suis*. *Streptococcus suis* SC19 cells were co-cultured with each inhibitor in TSB medium to the log-phase. The cells were harvested, washed, and subjected to Gram staining followed by observation using an optical microscope. **(C)** 30 chains of were randomly chosen from each Gram staining images of SC19 treated with Staurosporine, AT9283, APY29, and K252a, respectively. Next, numbers of cells per chain were counted, respectively, and the data were presented as the means ± standard errors (*n* = 30). Student’s *t*-test was used to analyze their chain length difference in GraphPad Prism 7 software, with the value of *p* < 0.001, ***. **(D)** *G. mellonella* larvae infection assay. *Galleria mellonella* larvae were inoculated with APY29 at 150, 100, 50, and 0 mg/kg•body weight after *S. suis* SC19 infection. The survival was recorded every 6 h for 48 h. The survival data were analyzed by log−rank test in GraphPad Prism 7 software, with a value of *p* < 0.01, **. The experiments were carried out two times independently under the same conditions.

Staurosporine, APY29, AT9283, and K252a were subjected to test for their antimicrobial activity against *S. suis* by measuring the growth of *S. suis* in the CDM in the presence of 50 μM of each inhibitor. It was shown that the growth was significantly inhibited by the presence of the inhibitors which was similar to that of the Δ*stk* strain (Figure 7A). Moreover, it was observed that *S. suis* cells showed chained morphology when treated with Staurosporine, APY29, AT9283, or K252a at 50 μM, resembling Δ*stk* (Figures 7B,C). We further evaluated the *in vivo* antimicrobial efficacy of the compounds using a *G. mellonella* larvae infection model. It was shown that the group treated with 150 mg/kg • body weight of APY29 displayed a significantly higher survival than treated with normal saline after infection with *S. suis* SC19. The results indicate that APY29 had potent antimicrobial activity *in vivo* (Figure 7D).

### Molecular modeling between ssSTK and the inhibitors

**Figure 8:**
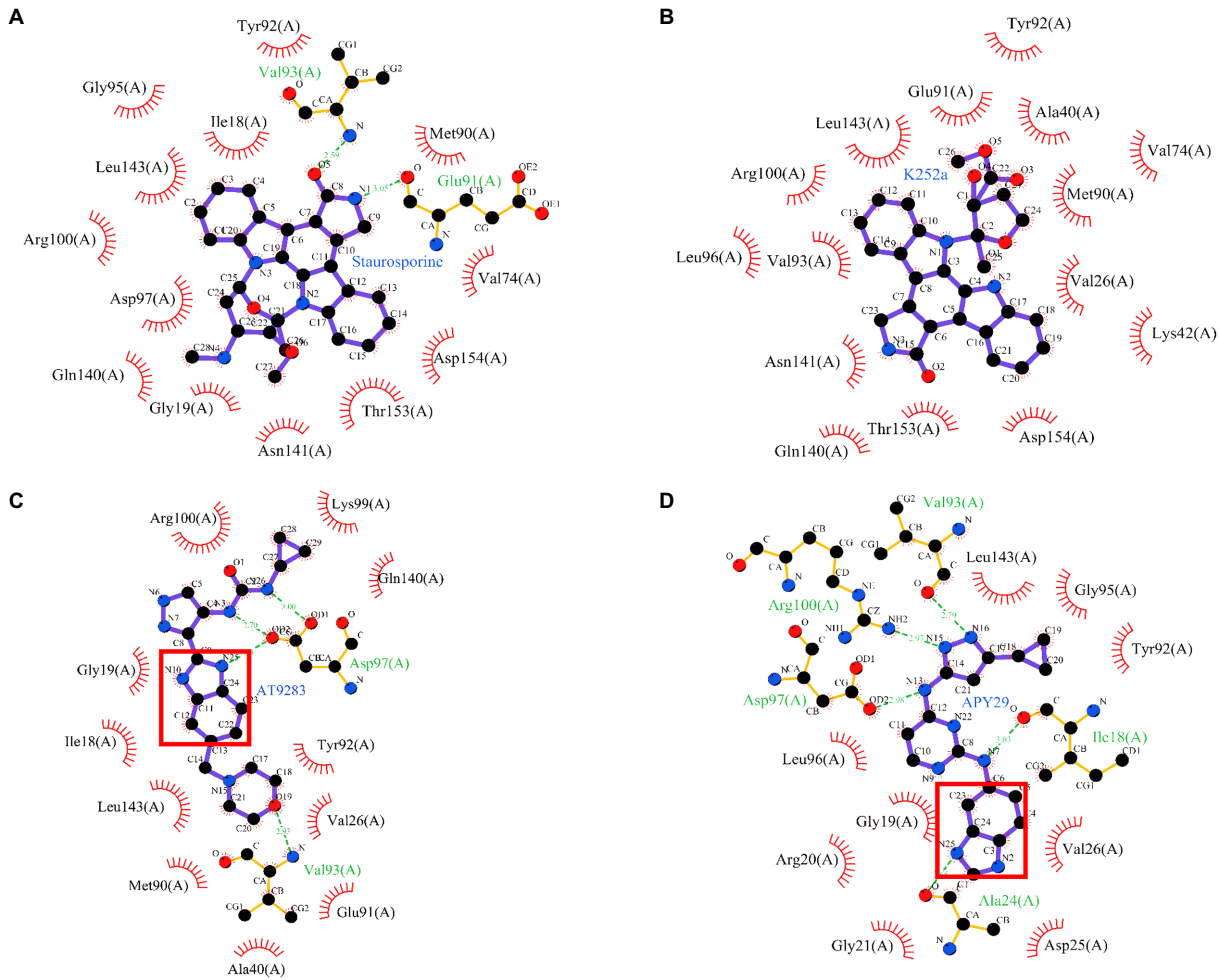
Molecular docking. Staurosporine **(A)**, K252a **(B)**, AT9283 **(C)**, and APY29 **(D)** were docked into the ATP-binding pocket of ssSTK using autodock4 software, respectively. The complex models were shown using Ligplus software (version 2.2.), Benzimidazole nucleus was marked by red box.

In order to investigate the binding modes between ssSTK and the inhibitors. These inhibitors were docked into the ATP-binding pocket of ssSTK, respectively. The lowest binding energy complex models suggested that these inhibitors interact with ssSTK *via* hydrogen bones and hydrophobic forces (Figure 8). It was suggested from the lowest binding complex models that the Val93 in the pocket of ssSTK could interact with Staurosporine, AT9283, APY29 and K252a (Figure 8), which may provide an insight into the design of ssSTK inhibitors based on the structure in the future. Additionally, it was shown in Figure 8D that the benzimidazole nucleus of APY29 interacted with ssSTK *via* hydrogen bond which may further explain the bioactivity difference between APY29 and VEGF2-2-IN-5 hydrochloride.

## Discussion

AMR has become a huge threat to public health worldwide (Samreen et al., 2021). Urgent actions are needed to take to deal with AMR. Despite several other strategies to control AMR, innovation on novel antimicrobials has been proposed as one of the most powerful approaches (Hutchings et al., 2019; Moo et al., 2020; Miethke et al., 2021). In this study, we show that autophosphorylation of the important bacterial kinase STK is involved in the regulation of growth, morphology, and pathogenicity of *S. suis*. Four compounds Staurosporine, AT9283, APY29, and K252a are identified as the inhibitors against ssSTK. They demonstrated effective antimicrobial activity in the CDM *in vitro*, among which APY29 showed *in vivo* efficacy against *S. suis* infection using a *G. mellonella* larvae infection model. Our results illustrated the feasibility of bacterial kinase ssSTK as an antimicrobial target and provide a good starting point for further antimicrobial drug development.

Considering the key role of protein phosphorylation modification in cellular functions, protein kinases have been deemed as attracting drug targets (Singh et al., 2017). So far, 71 kinase inhibitors have been approved by the U.S. Food and Drug Administration (FDA; Attwood et al., 2021). Most of them are applied for cancer treatment, such as Imatinib, an early identified Bcr-Abl kinase inhibitor for the treatment of chronic myelogenous leukemia (de Kogel and Schellens, 2007). In addition, several kinase inhibitors are developed for other diseases, such as tofacitinib for the treatment of rheumatoid arthritis (Dhillon, 2017). Although, protein kinases also play important roles in bacterial physiology, unfortunately, antibiotics targeting bacterial kinases have not yet been developed.

In bacteria, two-component systems, tyrosine kinase-phosphatase systems, and serine/threonine kinase-phosphatase systems comprise the main protein phosphorylation systems (Kobir et al., 2011; Mijakovic et al., 2016). Due to their critical roles in bacterial growth, metabolism, cell division, and pathogenesis, they have been regarded not only as global regulators but also promising anti-virulence or antimicrobial targets (Hirakawa et al., 2020; Nagarajan et al., 2021). Efforts have been made to screen inhibitors against these phosphorylation systems. Compound NH125 was shown as an inhibitor against several HKs including YycG, PhoQ, and EnvZ, and displayed bactericidal activity against *S. aureus*, *Bacillus subtilis,* and *S. pneumonia* (Yamamoto et al., 2000, 2001). Walkmycin C was identified to inhibit autophosphorylation of VicK, CiaH, and LiaS in *Streptococcus mutans* (Eguchi et al., 2011). Recently, Streptozotocin and floxuridine have been identified as inhibitors against the two-component system SaeRS of *S. aureus* and provided protection for mice against *S. aureus* (Yeo et al., 2018). By targeting the protein tyrosine phosphatase, inhibitor I-A09 has been identified and shown to inhibit the growth of *Mycobacterium tuberculosis* in host cells (Zhou et al., 2010).

Serine/threonine kinases, another important class of bacterial kinase, have recently been demonstrated to be involved in the regulation of critical physiological processes and virulence of several important bacterial pathogens, making them attractive drug targets (Manuse et al., 2016; Djorić et al., 2021). Recently, several inhibitors against STKs have been identified. Fernandez and his colleagues have identified Staurosporine and K252a as inhibitors against PknB of *M. tuberculosis* by autoradiography of SDS-PAGE analysis, which prevented mycobacterial growth (Fernandez et al., 2006). They have also conducted an *in silico* screening to discover PknB inhibitors and Mitoxantrone was identified as an ATP-competitive PknB inhibitor with potent antimicrobial activity (Wehenkel et al., 2006). Additionally, based on the susceptibility of *L. monocytogenes prkA* deletion mutant strain to *β*-lactams antibiotics, Schaenzer et al. have performed a primary screening for ceftriaxone adjuvants followed by an enzymatic inhibition assay, and confirmed Imidazopyridine aminofurazans as a specific inhibitor showing bioactivity against PrkA of *L. monocytogenes* rather than Stk1 of *S. aureus* (Schaenzer et al., 2017). Although several inhibitors against bacterial STKs have been discovered, none of them was permitted for antimicrobial application due to their toxicity or druggable defects. In this study, we proposed a novel strategy of STK inhibitors screening based on the growth defect of *S. suis stk* deletion mutant in the minimal medium and enzymatic inhibition assay, and identified several inhibitors against ssSTK and *S. suis* SC19 strain.

To investigate the structure–activity relationships (SARs), a series of indolocarbazole derivatives were tested for their inhibitory activity against ssSTK, and only Staurosporine and K252a showed potent inhibition. The bioactivity difference of the indolocarbazole derivatives is worthy of further analysis. Previous studies on SARs of indolocarbazole derivatives against PKCs, a family of eukaryotic serine/threonine kinases, revealed the important role of glycosidic linkage at N-12 and N-13 position of indolocarbazole in the bioactivity of these compounds (Wang et al., 2020). Our data also suggested that the glycosidic linkage improves the bioactivity of indolocarbazole against ssSTK. Additionally, the structural comparison of K252c with indolocarbazole showed that the pyrrole ring possibly facilitates the inhibition activity. However, the comparison of Staurosporine and Midostaurin suggested that N-methybenzamide substitution at C-4′ position of pyran ring reduces the bioactivity of indolocarbazole derivatives. The SARs analysis could provide insights into the structural modifications of indolocarbazole derivatives against ssSTK. Moreover, our results showed that Staurosporine and K252a displayed antimicrobial efficacy *in vitro*. However, they also showed toxicity to host cells which may be due to a lack of kinase selectivity (Lazarovici et al., 1996). Therefore, fragment-based optimization of Staurosporine and K252a may be helpful to resolve this problem.

The benzimidazole derivatives show various biological activities due to their structural resemblance with purine (Bansal et al., 2019). However, their antimicrobial activity has rarely been studied. AT9283 and APY29 shared a benzimidazole nucleus. Previous studies have identified AT9283 and APY29 as kinase inhibitors with anticancer and anti-apoptosis activities, respectively (Korennykh et al., 2009; Santo et al., 2011; Qi et al., 2012; Gu et al., 2021). In the current study, AT9283 and APY29 were demonstrated as inhibitors against ssSTK exerting antimicrobial activity against *S. suis*. The structure and activity comparison of APY29 and its analogue VEGFR-2-IN-5 revealed that the bioactivity of APY29 might be partly attributed to its benzimidazole nucleus. Additionally, the molecular docking models also show that the benzimidazole nucleus of APY29 or AT9283 interacts with ssSTK *via* hydrogen bond. Thus, discovering ssSTK inhibitors from benzimidazole derivatives is a promising strategy. Although the primary SAR of APY29 and AT9283 was analyzed, further structural optimizations for higher activity and lower toxicity are still needed.

## Conclusion

In conclusion, we demonstrate the feasibility of targeting the autophosphorylation ssSTK as a strategy for antimicrobial compounds screening and identified 4 compounds, Staurosporine, APY29, AT9283, and K252a, as inhibitors against ssSTK. These compounds showed potent inhibition of the growth of *S. suis* in the minimal medium and one of them showed *in vivo* efficacy against *S. suis* infection. Our study provides promising candidates for further antimicrobial development.

## Data availability statement

The original contributions presented in the study are included in the article/Supplementary material, further inquiries can be directed to the corresponding authors.

## Author contributions

HL and QiH: conceptualization. HL, QiaH, and LL: formal analysis. QiH and RZ: funding acquisition, project administration, supervision, and writing–review and editing. HL and TL: investigation. HL, ZY, and QiH: methodology. QiH: validation. HL: writing–original draft. All authors contributed to the article and approved the submitted version.

## Funding

This work was supported by the National Key Research and Development Plans of China (2018YFE0101600 and 2021YFD1800401).

## Conflict of interest

The authors declare that the research was conducted in the absence of any commercial or financial relationships that could be construed as a potential conflict of interest.

## Publisher’s note

All claims expressed in this article are solely those of the authors and do not necessarily represent those of their affiliated organizations, or those of the publisher, the editors and the reviewers. Any product that may be evaluated in this article, or claim that may be made by its manufacturer, is not guaranteed or endorsed by the publisher.

## Footnotes

^1^ https://zhanglab.ccmb.med.umich.edu/I-TASSER/
