# Supplementary material for: Inhibitors targeting the autophosphorylation of serine/threonine kinase of Streptococcus suis show potent antimicrobial activity
Source: Front Microbiol. 2022 Sep 2;13:990091. doi: 10.3389/fmicb.2022.990091 (PMC9478340; doi:10.3389/fmicb.2022.990091)
Supplement: Supplementary file 1 [file Table_1.docx]

Supplementary Material

**Supplementary Table S1. Bacterial strains and plasmids used in this study**

| Name | Description | Reference or source |
| --- | --- | --- |
| Strains |  |  |
| *S. suis* SC19 | *S. suis* serotype 2 | Laboratory stock |
| *S. suis* Δ*stk* | The isogenic *stk* deletion mutant of SC19 | Laboratory stock |
| *S. suis* CΔ*stk* | The complementary strain | Laboratory stock |
| *S. suis Δstk::stk^T167A-S175A^* | *S. suis* Δ*stk* strain contains pSET2-*pro*-*stk^T167A-S175A^* plasmid | This study |
| *E. coli* DH5α | *E. coli* for the molecular clone | Laboratory stock |
| *E. coli* BL21(DE3) | *E. coli* for the expression of proteins | Laboratory stock |
| Plasmids |  |  |
| pET28a-*stk^KD^* | Plasmid for the expression of ssSTK, Kan^r^ | This study |
| pET28a-*stk_KD-T167A_* | Plasmid for the expression of ssSTK-T167A, Kan^r^ | This study |
| pET28a-*stk^KD-T169A^* | Plasmid for the expression of ssSTK-T169A, Kan^r^ | This study |
| pET28a-*stk^KD-T175A^* | Plasmid for the expression of ssSTK-S175A, Kan^r^ | This study |
| ^a^pSET2-*pro*-*stk^T167A-S175A^* | Plasmid for the complementary, Spc^r^ | This study |

a, the pSET2-based recombinant plasmid to encode the variant ssSTK (T167A-S175A point mutations) in *S. suis*

**Supplementary Table S2. The composition of chemically defined medium (CDM)**

| Component | Concentration (mg/L) | Component | Concentration (mg/L) |
| --- | --- | --- | --- |
| FeSO_4_ ^.^ 7H_2_O | 5 | L-Tyrosine | 100 |
| Fe(NO_3_)_2_ ^.^ 9H_2_O | 1 | L-Valine | 100 |
| K_2_HPO_4_ | 200 | *p*-Aminobenzoic acid | 0.2 |
| KH_2_PO_4_ | 1000 | Biotin | 0.2 |
| MgSO_4_ ^.^ 7H_2_O | 700 | Folic acid | 0.8 |
| MnSO_4_ | 5 | Niacinamide | 1 |
| DL-Alanine | 100 | β-Nicotinamide adenine dinucleotide | 2.5 |
| L-Arginine | 100 | Pantothenate calcium salt | 2 |
| L-Aspartic acid | 100 | Pyridoxal | 1 |
| L-Cystine | 50 | Pyridoxamine dihydrochloride | 1 |
| L-Glutamic acid | 100 | Riboflavin | 2 |
| L-Glutamine | 200 | Thiamine hydrochloride | 1 |
| Glycine | 100 | Vitamin B_12_ | 0.1 |
| L-Histidine | 100 | Glucose | 10000 |
| L-Isoleucine | 100 | Adenine | 20 |
| L-Leucine | 100 | Guanine hydrochloride | 20 |
| L-Lysine | 100 | Uracil | 20 |
| L-Methionine | 100 | CaCl_2_ ^.^ 6H_2_O | 10 |
| L-Phenylalanine | 100 | NaC_2_H_3_O_2_ ^.^ 3H_2_O | 4500 |
| L-Proline | 100 | L-Cysteine | 500 |
| Hydroxy-L-proline | 100 | NaHCO_3_ | 2500 |
| L-Serine | 100 | NaH_2_PO_4_ ^.^ H_2_O | 3195 |
| L-Threonine | 200 | Na_2_HPO_4_ | 7350 |
| L-Tryptophan | 100 |  |  |

*All the chemicals were purchased from Bioshrpe, Bioforxx or Solarbio.

**Supplementary Table S3. Primers used in this study**

| Premiers | Sequence (5’-3’) |
| --- | --- |
| STK^KD^-F | GTGCCGCGCGGCAGCCATATGATGATTCAAATCGGTAAGATC |
| STK^KD^-R | GTGGTGGTGGTGGTGCTCGAGTTGGGAGAGTTTCGGCAA |
| STK^KD-T167A^-F | CAGTTGCCTTTGCTGAAACGAGTTTGGCACAAACCAACTCC |
| STK^KD-T167A^-R | GGAGTTGGTTTGTGCCAAACTCGTTTCAGCAAAGGCAACTG |
| STK^KD-T169A^-F | CAGTTGCCTTTGCTGAAACGAGTTTGACACAAGCCAACTCC |
| STK^KD-T169A^-R | GGAGTTGGCTTGTGTCAAACTCGTTTCAGCAAAGGCAACT |
| STK^KD-S175A^-F | CCAACTCCATGTTGGGTGCTGTTCACTATCTTTCACCTGA |
| STK^KD-S175A^-R | TCAGGTGAAAGATAGTGAACAGCACCCAACATGGAGTTG |
| HR-Pro-STK-F | AGGTCGACTCTAGAGGATCCACTCATAATCTCTACCAC |
| HR-Pro-STK-R | AAACGACGGCCAGTGAATTCTTATTGTCCGCTACCTGTT |
| Pro-STK-F | GGAGATGAGAACACGAATATGATTCAAATCGGTAAGAT |
| Pro-STK-R | ATCTTACCGATTTGAATCATATTCGTGTTCTCATCTCC |

**Supplementary Table S4.** **Inhibition rate of compounds against *S. suis* SC19**

| Drug name | Inhibition against *S. suis* SC19 |
| --- | --- |
| Perifosine | 99.50 % |
| Afuresertib (hydrochloride) | 94.30 % |
| CX-6258 | 90.90 % |
| Sitravatinib | 90.00 % |
| WZ4002 | 97.60 % |
| Foretinib | 87.40 % |
| FRAX486 | 98.90 % |
| CHZ868 | 92.40 % |
| Cediranib (maleate) | 100.00 % |
| Masitinib | 94.10 % |
| DDR1-IN-1 | 98.00 % |
| GSK461364 | 92.40 % |
| IKK 16 | 91.10 % |
| A419259 (trihydrochloride) | 96.50 % |
| CEP-37440 | 100.00 % |
| Nazartinib | 97.40 % |
| Epertinib (hydrochloride) | 86.60 % |
| A-674563 (hydrochloride) | 90.50 % |
| IKK-IN-1 | 95.20 % |
| Brivanib (alaninate) | 86.80 % |
| YLF-466D | 98.40 % |
| Kira8 | 98.40 % |
| B-Raf IN 1 | 88.80 % |
| PKC-theta inhibitor | 92.40 % |
| Voruciclib (hydrochloride) | 100.00 % |
| ALK inhibitor 1 | 93.40 % |
| BMS-509744 | 90.80 % |
| MRX-2843 | 100.00 % |
| GW2580 | 89.60 % |
| MK2-IN-1 (hydrochloride) | 100.00 % |
| Honokiol | 100.00 % |
| ENMD-2076 | 100.00 % |
| HG-14-10-04 | 100.00 % |
| P110δ-IN-1 | 100.00 % |
| Oridonin | 100.00 % |
| LY2608204 | 98.10 % |
| (+)-Usnic acid | 100.00 % |
| 3-Bromopyruvic acid | 96.60 % |
| DEL-22379 | 100.00 % |
| NH125 | 86.70 % |
| BIX 02565 | 97.30 % |
| MK8722 | 85.10 % |
| Asciminib | 90.70 % |
| mTOR inhibitor-1 | 97.30 % |
| Bemcentinib | 96.30 % |
| Staurosporine | 91.70 % |
| Mutated EGFR-IN-1 | 97.60 % |
| (2S,3R)-Voruciclib (hydrochloride) | 98.00 % |
| MK-3903 | 100.00 % |
| PD0166285 | 100.00 % |
| DCP-LA | 97.20 % |
| CP-547632 | 92.00 % |
| Derazantinib | 97.20 % |
| CDK9-IN-2 | 96.30 % |
| NVP-2 | 100.00 % |
| Skepinone-L | 99.10 % |
| GW284543 | 81.80 % |
| Tarloxotinib (bromide) | 95.80 % |
| GSK2110183 analog 1 (hydrochloride) | 100.00 % |
| (1S,3R,5R)-PIM447 (dihydrochloride) | 90.50 % |
| HG6-64-1 | 99.30 % |
| PIM-447 (dihydrochloride) | 92.60 % |
| SK1-IN-1 | 100.00 % |
| BI-4464 | 99.40 % |
| BDP5290 | 88.80 % |
| Pelitinib | 94.80 % |
| D-erythro-Sphingosine | 100.00 % |
| Src Inhibitor 1 | 96.00 % |
| BI-78D3 | 99.00 % |
| NSC 228155 | 90.00 % |
| GW 5074 | 89.70 % |
| GZD824 | 99.00 % |
| CX-6258 (hydrochloride hydrate) | 94.40 % |
| Carnosol | 94.40 % |
| CGK733 | 91.70 % |
| ARQ 531 | 91.40 % |
| Refametinib | 98.30 % |
| SEL120-34A (monohydrochloride) | 100.00 % |
| Chelerythrine Chloride | 100.00 % |
| Afuresertib | 97.90 % |
| TBB | 92.20 % |
| Edicotinib | 99.90 % |
| FF-10101 | 89.00 % |
| AT9283 | 85.90 % |
| Ralimetinib dimesylate | 97.30 % |
| Degrasyn | 94.10 % |
| BMS-345541 (hydrochloride) | 100.00 % |
| NVP-TAE 684 | 96.80 % |
| Ensartinib hydrochloride | 86.10 % |
| TAK-285 | 96.80 % |
| SB-590885 | 93.60 % |
| BI-847325 | 89.60 % |
| SC66 | 98.40 % |
| XMD17-109 | 100.00 % |
| BAY 11-7082 | 83.70 % |
| Avitinib (maleate) | 96.00 % |
| Gandotinib | 93.40 % |
| APY29 | 92.90 % |
| PRT062607 (Hydrochloride) | 100.00 % |
| BS-181 | 100.00 % |
| PQ401 | 91.90 % |
| IMD-0354 | 99.90 % |
| Dovitinib | 99.30 % |
| Dovitinib (lactate) | 98.90 % |
| AG 555 | 86.00 % |
| WP1066 | 100.00 % |
| GNF-5 | 100.00 % |
| SGI-1776 | 100.00 % |
| Olmutinib | 100.00 % |
| R1530 | 100.00 % |
| URMC-099 | 100.00 % |
| IPA-3 | 98.70 % |
| 1-NM-PP1 | 100.00 % |
| TDZD-8 | 100.00 % |
| SKI II | 100.00 % |
| GSK 3 Inhibitor IX | 81.60 % |
| WH-4-023 | 95.60 % |
| PHA-665752 | 95.00 % |
| WZ4003 | 99.60 % |
| CHIR-124 | 100.00 % |
| Isobavachalcone | 100.00 % |


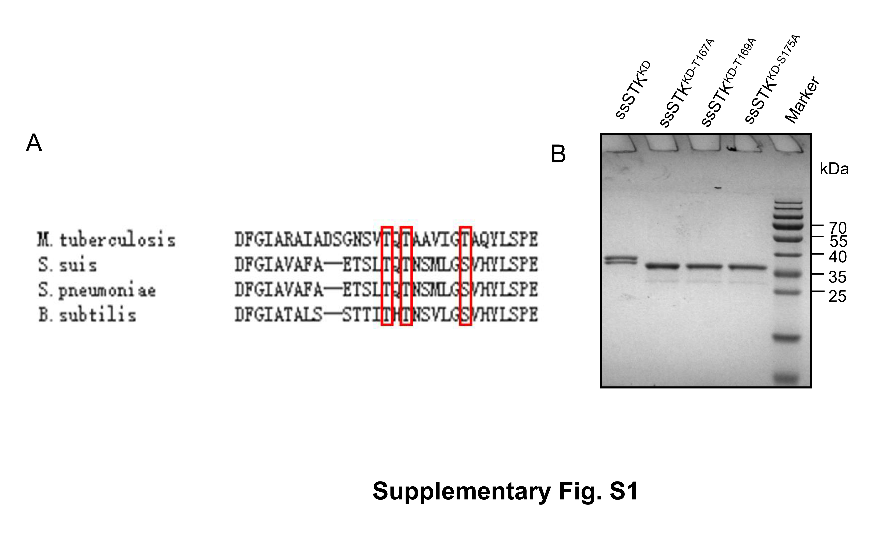


Supplementary Fig. S1. Prediction and identification of autophosphotylation sites of ssSTK. (A) Multiple sequence alignment of STKs from different bacteria. Multiple sequence alignment was generated by using the Clustal Omega server based on amino acid sequence of STKs activation loop from each indicated bacterium. The potential autophosphorylation sites are shown in red box. (B) After phosphorylation reaction, the samples including ssSTK^KD^ and its derivatives were analyzed by SDS-PAGE.


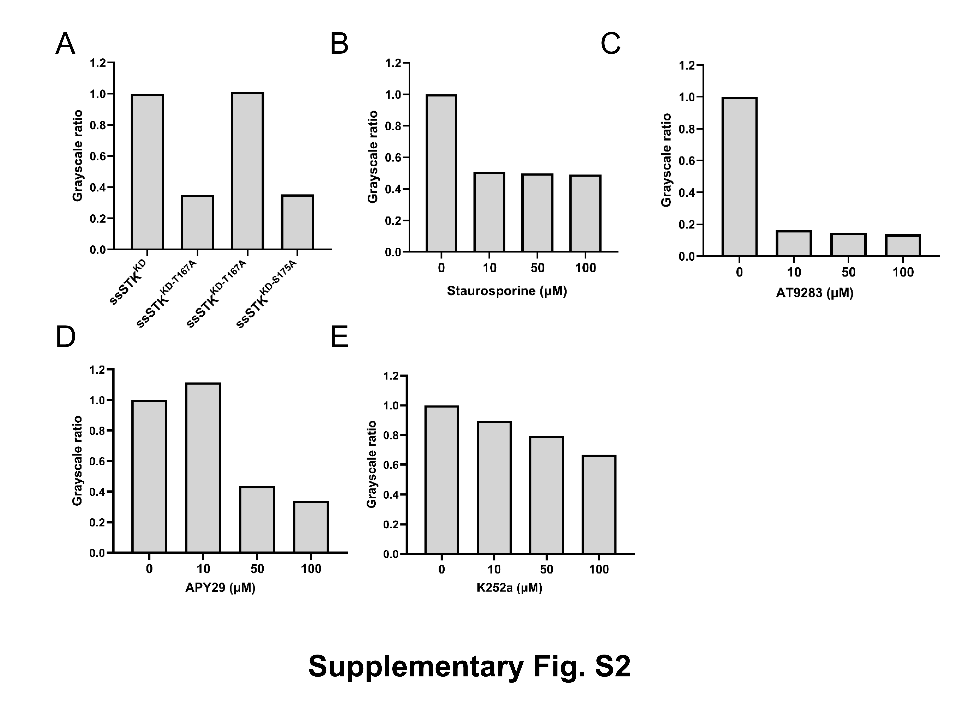


Supplementary Fig. S2. Analysis of grayscale for bands of Western blot. The Grayscale values of all bands in Western blot images including (A) Fig. 1C, (B) Fig. 4A, (C) Fig. 4B, (D) Fig. 4C, (E) Fig. 4D were measured by Image lab software, respectively. Grayscale ratio were calculated by normalizing kinase phosphorylated levels based on the first band in all images, respectively.
